# Mold and Endotoxin Levels in the Aftermath of Hurricane Katrina: A Pilot Project of Homes in New Orleans Undergoing Renovation

**DOI:** 10.1289/ehp.9258

**Published:** 2006-08-24

**Authors:** Ginger L. Chew, Jonathan Wilson, Felicia A. Rabito, Faye Grimsley, Shahed Iqbal, Tiina Reponen, Michael L. Muilenberg, Peter S. Thorne, Dorr G. Dearborn, Rebecca L. Morley

**Affiliations:** 1 Department of Environmental Health Sciences, Mailman School of Public Health, Columbia University, New York, New York, USA; 2 National Center for Healthy Housing, Columbia, Maryland, USA; 3 Department of Epidemiology and; 4 Department of Environmental Health, Tulane School of Public Health and Tropical Medicine, New Orleans, Louisiana, USA; 5 Department of Environmental Health, University of Cincinnati, Cincinnati, Ohio, USA; 6 Department of Environmental Health, Harvard School of Public Health, Boston, Massachusetts, USA; 7 Department of Occupational & Environmental Health, The University of Iowa, College of Public Health, Iowa City, Iowa, USA; 8 Department of Environmental Health Sciences, Case Western Reserve University, Cleveland, Ohio, USA

**Keywords:** endotoxin, flood, fungi, mold, Hurricane Katrina, New Orleans, remediation, respirators

## Abstract

**Background:**

After Hurricane Katrina, many New Orleans homes remained flooded for weeks, promoting heavy microbial growth.

**Objectives:**

A small demonstration project was conducted November 2005–January 2006 aiming to recommend safe remediation techniques and safe levels of worker protection, and to characterize airborne mold and endotoxin throughout cleanup.

**Methods:**

Three houses with floodwater lines between 0.3 and 2 m underwent intervention, including disposal of damaged furnishings and drywall, cleaning surfaces, drying remaining structure, and treatment with a biostatic agent. We measured indoor and outdoor bioaerosols before, during, and after intervention. Samples were analyzed for fungi [culture, spore analysis, polymerase chain reaction (PCR)] and endotoxin. In one house, real-time particle counts were also assessed, and respirator-efficiency testing was performed to establish workplace protection factors (WPF).

**Results:**

At baseline, culturable mold ranged from 22,000 to 515,000 colony-forming units/m^3^, spore counts ranged from 82,000 to 630,000 spores/m^3^, and endotoxin ranged from 17 to 139 endotoxin units/m^3^. Culture, spore analysis, and PCR indicated that *Penicillium, Aspergillus, and Paecilomyces* predominated. After intervention, levels of mold and endotoxin were generally lower (sometimes, orders of magnitude). The average WPF against fungal spores for elastomeric respirators was higher than for the N-95 respirators.

**Conclusions:**

During baseline and intervention, mold and endotoxin levels were similar to those found in agricultural environments. We strongly recommend that those entering, cleaning, and repairing flood-damaged homes wear respirators at least as protective as elastomeric respirators. Recommendations based on this demonstration will benefit those involved in the current cleanup activities and will inform efforts to respond to future disasters.

Hurricane Katrina, which occurred on 29 August 2005, caused the breach of several levees surrounding New Orleans, Louisiana, and left over 75% of the city underwater. A storm surge from Hurricane Rita several weeks later reflooded many areas of the city. In the aftermath of these disasters, homes were submerged in flood water for several summer weeks, resulting in severe mold and bacterial growth, both of which can lead to a panoply of respiratory health effects [Institute of Medicine (IOM) 2004]. Although several studies have assessed markers of mold and bacteria in damp and water-damaged homes (IOM 2004), there is a dearth of literature documenting the levels of these agents in homes that received sustained flooding (Ross et al. 2000).

New Orleans and the surrounding communities currently face difficult decisions regarding whether or not to demolish large numbers of damaged homes. Many factors will be taken into consideration when those decisions are made, including the question of whether mold and bacteria can be safely and affordably removed from the flooded homes prior to building restoration. On 27 October 2005, the National Center for Healthy Housing (NCHH) convened a meeting to examine options for flood cleanup procedures and air sampling. Attendees included building materials scientists, mold remediation experts, members of community-based organizations, industrial hygienists, clinicians, housing policy makers, and environmental epidemiologists from across the country (including areas affected by Hurricane Katrina). The attendees drew on a body of guidance documents for flood cleanup and mold remediation [American Red Cross and Federal Emergency Management Agency 1992; Centers for Disease Control and Prevention (CDC) 2005; Louisiana State University 2005; New York City Department of Health and Mental Hygiene 2002; U.S. Environmental Protection Agency (EPA) 2001).

The overall purpose of this demonstration was to assess the efficacy of flood cleanup procedures in three houses in New Orleans, which sustained between 0.3 and 1.8 m of flood damage from Hurricanes Katrina and Rita. Three specific objectives of the demonstration were *a*) to make recommendations for safe reentry into a home and safe removal of flood-damaged furnishings and building materials, including levels of worker protection; *b*) to characterize the distribution of airborne mold and endotoxin (a cell wall component of gram-negative bacteria) throughout all phases of the cleanup; and *c*) to comment on the practicality of the overall cleanup process. To this end, Enterprise Community Partners and the NCHH, in collaboration with Neighborhood Housing Services of New Orleans and several partners from academic institutions, studied these issues as part of the demonstration project, which was conducted between November 2005 and January 2006.

## Materials and Methods

### Selection of homes

Three single-family houses in the Gentilly district of New Orleans were selected to participate in the project. Most of the areas in Gentilly lie 0.5–4 m below sea level [Greater New Orleans Community Data Center (GNOCDC) 2006a]. Eighty-five percent of the total households in this area suffered some degree of damage, and 71% of households were severely damaged or destroyed by flooding from Hurricane Katrina (GNOCDC 2006b).

To be included in the demonstration, a house had to be flooded with < 2 m of water, structurally sound, and located in an area likely to be rebuilt. Owners were required to have flood insurance deemed adequate to rebuild their house and intentions to rebuild. The first house was selected when an employee of a New Orleans–based nonprofit housing organization volunteered his house for the project. Neighborhood Housing Services, a chartered member of NeighborWorks America (Washington, DC), identified two additional homeowners whose houses met the inclusion criteria.

At house 1, the homeowner selected the contractor to complete the work. At houses 2 and 3, Neighborhood Housing Services of New Orleans selected the contractors and supervised the activities, with support from the NCHH and Urban Renovation Consultants. Urban Renovation Consultants and the NCHH, as well as its national advisory work group, developed the construction specifications used at the three houses.

### Personal protective equipment

All workers and supervisors wore N100 filtering facepiece respirators, nonwoven polypropylene disposable coveralls, and gloves during the property inspection. During property removal and deconstruction, some workers also used half-face HEPA (high-efficiency particulate air) filter air-purifying respirators (some equipped with organic solvent filters) and goggles.

### Baseline inspection

The supervisors visually inspected each house for roof leakage, standing water, and the extent of mold on walls, cabinets, floors, doors, trim, appliances, equipment, and heating ventilation/air conditioning ductwork. The moisture content of the wood studs was tested using a moisture meter with a probe (J-LITE Moisture Meter, Delmhorst Instrument Company, Towaco, NJ); moisture readings > 22% were considered saturated, and those < 15% were considered dry (U.S. Department of Agriculture 1999). The supervisors determined whether electrical service, if operable, was safe and verified that there were no water or gas leaks.

In house 1, surfaces were relatively dry at the time of the demonstration, with no standing water. Possessions with visual mold and limited value to the owner of house 1 were discarded. Other hard surface possessions were cleaned outside with a bleach–detergent solution and then packed in boxes and moved to the second floor of the house. House 2 had been closed since Hurricane Katrina and was still relatively wet at the start of the project, with standing water, wet furnishings, and plaster and drywall surfaces saturated with water. In house 3, personal belongings, furniture, and lower cabinets had been removed by the time of the initial inspection. The carpet and padding were wet, and the drywall was saturated within 0.3 m of the floor. Details of the baseline conditions and demonstration activities are listed in Table 1.

### Deconstruction

Deconstruction at all houses included removing carpet or otherwise clearing floors down to the finished flooring and removing insulation, nails on studs, and lower cabinets, if present. At house 1, bathroom toilets, sinks, and bathtubs were also removed. At house 2, workers also removed upper cabinets.

### Cleaning and sanitizing

Workers cleaned and sanitized all three houses with a combination of dry-cleaning and wet-cleaning steps. Workers conducted dry cleaning by bristle brushing studs and other framing members to remove visible mold growth and then vacuuming the same surfaces with a HEPA filter attachment. Wet cleaning was completed by using sponge mops and hand sponges to wash down surfaces with a non-trisodium phosphate solution of sodium sesquicarbonate detergent and 15% dilution of household chlorine bleach. In all three houses, dry cleaning was conducted before wet cleaning. In house 2, all surfaces including all framing members were wet cleaned. In houses 1 and 3, workers wet cleaned only hard, nonporous surfaces (e.g., tubs, sinks, toilets, tile flooring).

### Biostat treatments

After cleaning, the three houses received biostat treatment with one of two borate formulations to prevent future emergence of mold. House 1 was treated with Bora-Care (Nisus Corp., Rockford, TN); house 2 was treated with Bora-Care in half the house and Termite Prufe (Copper Brite, Inc., Santa Barbara, CA) in the other half; and house 3 was treated with Termite Prufe. The active ingredient in both Bora-Care and Termite Prufe is disodium octoborate tetra-hydrate. Both products are fungicides registered by the U.S. EPA. Bora-Care was mixed with water (1:3 or 1:4) and sprayed onto all lumber sheathing to the point of wetness in conformance with the manufacturer’s instructions. Termite-Prufe (0.45 kg powder to 3.8 L water) was applied to all exposed interior surfaces at 18.7 m^2^ per 3.8 L, working from ceiling to floor. The two formulations differ significantly in cost after mixing (Bora-Care was 2.5 times more expensive than Termite Prufe).

### Drying

Windows in houses 2 and 3 were left open to allow cross-ventilation to dry the moisture introduced by the biostat treatments. All possessions had been removed from these houses, so security was not a concern. In house 1, some possessions were stored on the second floor, thus windows were closed, but the upstairs ceiling fan was used. No mechanical dehumidification was used in any of the houses.

### Air sampling

We conducted air sampling during three time points for each home: *a*) before intervention (i.e., day 1), *b*) during intervention (i.e., day 1 or 2), and *c*) after intervention (i.e., day 36, 23, and 15, respectively, for houses 1, 2, and 3). The preintervention sampling for house 1 occurred 1 day after some items were removed from the house, but before work commenced on the second day. For houses 2 and 3, the preintervention sampling occurred either 1 day or a few hours before demonstration commenced. Intervention sampling occurred within a few hours of removal of mold-laden drywall. Postintervention sampling occurred between 1 and 2 weeks after borate solution was applied. Table 2 summarizes the air sampling activities conducted in each home.

#### Indoor samples

Using battery-operated air sampling pumps (AirChek 2000; SKC, Inc., Eighty Four, PA), we sampled the living room air for 20 min at a flow rate of 2.5 L/min with 2.0-μm pore Teflon filters (Omega Specialty, Inc., Chelmsford, MA) housed in 37-mm cassettes. In some houses, additional samples were collected upstairs (house 1) and in the bedroom (house 3). The pumps were attached to a tripod 1.2–1.35 m above the floor, with the sampling cassette open to the center of the room. Using high-flow (15 L/min) air sampling pumps (Environmental Monitoring Systems, Inc., Charleston, SC), fungal spores were collected in the living room air using BioCell impaction cassettes (GrafTech, Inc., Brighton, MI) for 2 min pre- and postintervention, and 1 min during intervention. Lack of electricity precluded postintervention indoor and outdoor sampling with the high-flow pumps for houses 2 and 3. Sampling trains were pre- and post-calibrated, and the average flow rate was used in calculations of volume of air sampled.

Particle counts were measured in real-time for house 3 (before and during intervention) using an optical particle counter (model 1.108; Grimm Technologies, Douglasville, GA). This instrument measured the number of particles in 15 size ranges, from 0.3 to 20.0 μm. We used an averaging time of 1 min.

We performed respirator-efficiency testing by measuring workplace protection factor (WPF) against fungal spores collected in house 3. The institutional review board of The University of Cincinnati approved this sampling protocol, and informed written consent was obtained by the university investigator who wore the respirators. We tested two types of respirators: a disposable N-95 filtering face-piece respirator (model 8110; 3M, St. Paul, MN) and an elastomeric half-facepiece respirator (North 5500-30 with North filter cartridge gas and vapor with P100 particulate filter; North Safety Products, Cranston, RI). The experimental protocols and the N-95 filtering facepiece respirator were as described in an agriculture study by Lee et al. (2005). In brief, the subject was fit-tested before the experiment using the Portacount method (TSI, Inc., St. Paul, MN). The subject wore the test respirator, which was connected to a newly developed personal sampling setup, for 30 min. In the sampling setup, fungal spores were collected from inside and outside the respirator onto polycarbonate filters. Concentration of fungal spores (spores per cubic meter) was determined through microscopic counting of the filters (Adhikari et al. 2003; Lee S-A et al. 2006). WPF was calculated by dividing the fungal spore concentration inside the respirator by that outside the respirator near the subject’s breathing zone. The test was repeated twice with the N-95 respirator and four times with the elastomeric respirator.

#### Outdoor samples

To collect outdoor samples, we placed the battery-operated AirChek 2000 pumps 3 m outside the front door for collection of one preintervention sample for house 3, one sample during the intervention for house 2, and postintervention samples for all three homes. We also collected samples before and during intervention using the high-flow pumps placed at 3 and 10 m outside the front door. Also, some outdoor samples were not collected during inclement weather because of concerns regarding technician safety and equipment damage.

#### Field blanks

On sampling days when intervention occurred, we collected a blank BioCell to assess fungal spores that could have been on the collection media before sampling, that were introduced in the moments before starting and after stopping the pump, and during sample transport. The BioCell cassette was opened for 1 min in the house (without attachment to a sampling pump), and then sealed. All blanks were negative for fungal spores.

### Analytical methods

#### Culture of fungi

Teflon filters were placed in 5 mL pyrogen-free water with 0.05% Tween 20 in sterile plastic tubes and shaken for 1 hr at 25°C. Samples were vortexed, and 100-μL aliquots (undiluted, 1:10, and 1:100 dilution) were spread-plated onto two types of culture media: dichloran glycerol and malt extract agar. Culture plates were incubated at two different temperatures: 25°C for 7–10 days or 37°C for 2–3 days. For each type of media and each temperature, the plates with nearest 10–30 colonies were counted. Culturable fungi were reported in colony-forming units (CFU).

#### Fungal spore counts

We analyzed fungal spores by direct microscopic examination of 27% of the impaction surface using transverse scans across the spore deposit at 400× magnification. Larger, infrequently recovered spore types were counted by scanning the full impaction surface at 200× magnification. In situations with very high spore concentrations, where resulting spore densities were extreme, fewer transverse scans were performed using a 120-μm reticule (for example, 0.017% of the deposit was scanned in one sample). Most spores were identified by genus; *Aspergillus, Penicillium, Eurotium*, and *Paecilomyces* are difficult to discern by this method and were grouped into one category.

#### Polymerase chain reaction (PCR)

Aliquots of the Teflon filter extracts were analyzed by P & K Microbiological Services, Inc. (Cherry Hill, NJ) for species-specific quantification using quantitative PCR (qPCR) following the methods previously described by Haugland et al. (2004) and Vesper et al. (2004). Reference controls were used as positive quantitative controls. Independent qPCR analyses were performed using primers and probes validated specifically for the 23 species/species groups of interest: *Acremonium strictum, Alternaria alternata, Aspergillus flavus/oryzae, Aspergillus fumigatus, Aspergillus niger, Aspergillus ochraceus, Aspergillus sydowii, Aspergillus ustus, Aspergillus versicolor, Eurotium (Aspergillus) amstelodami, Chaetomium globosum, Cladosporium cladosporioides, Memnoniella echinata, Paecilomyces variotii, Penicillium aurantiogriseum, Penicillium brevicompactum, Penicillium chrysogenum, Penicillium purpurogenum, Penicillium variabile, Scopulariopsis brevicaulis/fusca, Stachybotrys chartarum, Trichoderma viride/koningii,* and *Ulocladium botrytis.*

#### Endotoxin

Aliquots of the Teflon filter extracts were analyzed for endotoxin by the kinetic chromogenic *Limulus* amebocyte lysate assay (Thorne 2000; Vojta et al. 2002). Levels of endotoxin were reported in endotoxin units (EU).

## Results

### Comparison of levels before, during, and after intervention

**Figure 1:**
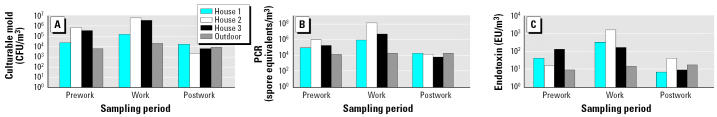
Results of filter samples from the three houses using three different analytical methods: total culturable mold (*A*; results are shown only for those grown on malt extract agar and cultured at 25°C), PCR (*B*), and endotoxin (*C*). Work for house 1 is the average of two measurements (upstairs and downstairs); outdoor prework is represented only by house 3; outdoor work is represented only by house 2; and outdoor postwork represents the average of the three houses.

**Figure 2:**
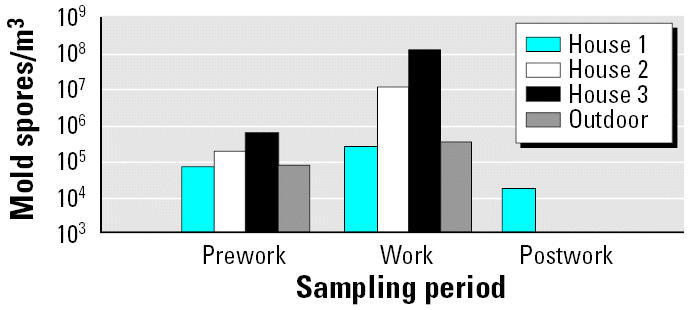
Results of mold spore counts. Only house 1 had an inside postwork measurement. Outdoor pre-work and work levels represent the average of the measurements for the three houses. Spore counts for house 1 decreased 77.6% between prework and postwork periods.

The baseline levels of mold and endotoxin, some of which varied by orders of magnitude within homes, are shown in Table 3. The preintervention samples for house 1 were taken after some belongings had been removed the previous day, yet the baseline levels were still generally below those of the other two houses. What is clear from Figure 1 is that postintervention levels of all bioaerosols in each house tended to be lower than at pre-intervention, except endotoxin in house 2, which was moderately elevated, and culturable mold in house 1, which had postintervention levels similar to those collected preintervention. Because generators were not available at all sampling periods at houses 2 and 3, several mold spore count measurements could not be collected. However, house 1 had spore measurements from all three time points, and the pattern was similar to that of the culturable fungi, PCR, and endotoxin results; levels increased during work and then decreased after intervention (Figure 2).

### Differences and similarities in fungal taxa profiles

**Figure 3:**
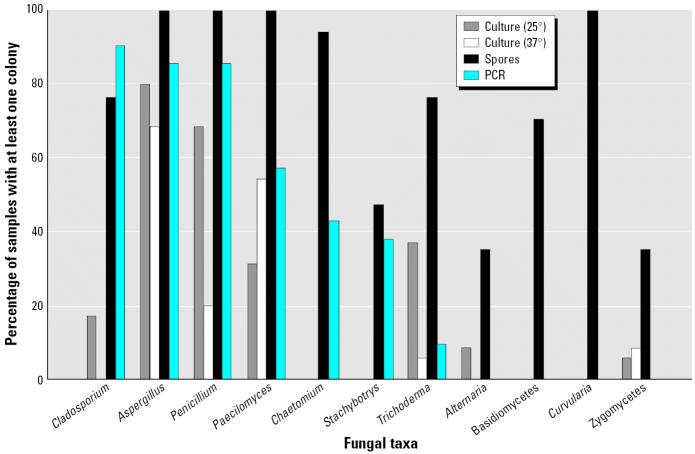
Frequency of fungal taxa in samples of indoor and outdoor air from the three houses and during different timepoints grouped together. The sample size limited interpretation when stratifying by location and timepoints. Culture at 25°C (*n* = 35); culture at 37°C (*n* = 35); spore counting (*n* = 17); and PCR (*n* = 21).

Interpretations of the predominant fungi recovered in the air samples differ depending upon the type of analysis. As shown in Figure 3, *Aspergillus, Paecilomyces*, and *Penicillium* were among the most frequently detected fungal taxa determined by all three methodologies (culture, PCR, and spore counting). Most of the spore count was due to *Aspergillus/Pencillium* spores. In house 1, *Stachybotrys* was recovered in the living room and in outdoor air during the intervention, but decreased to nondetectable levels by the postintervention sampling visit. *Cladosporium* was not frequently recovered from culture; however, it was frequently detected by PCR and spore counts, but at low concentrations relative to *Aspergillus* and *Penicillium*. In addition, spores of *Curvularia* and the Basidiomycota (including rusts, smuts, and basidiospores) were identified by microscopy. Neither PCR nor culture methods used in this analysis had the ability to measure spores of the Basidiomycota. Culture at 25°C and spore counting showed that *Trichoderma* was commonly recovered in the indoor air samples. A PCR probe for *Trichoderma virde/koningii* had a low recovery, suggesting that other methods may have been detecting another common *Trichoderma* species such as *Trichoderma harzianum*.

### Particle counts

**Figure 4:**
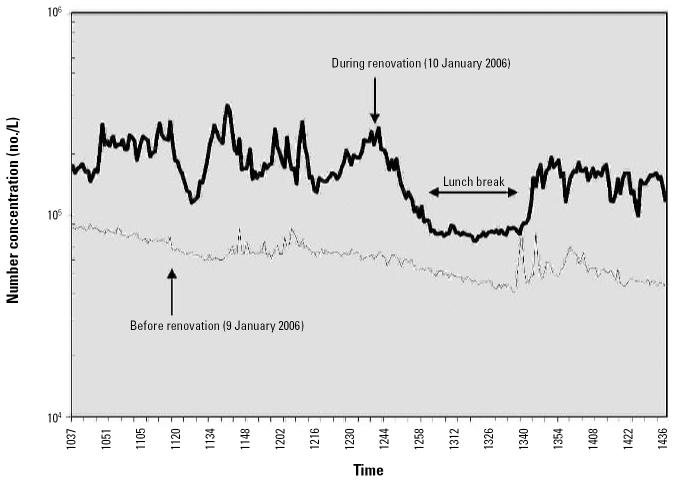
Total particle concentrations before and during the renovation.

**Figure 5:**
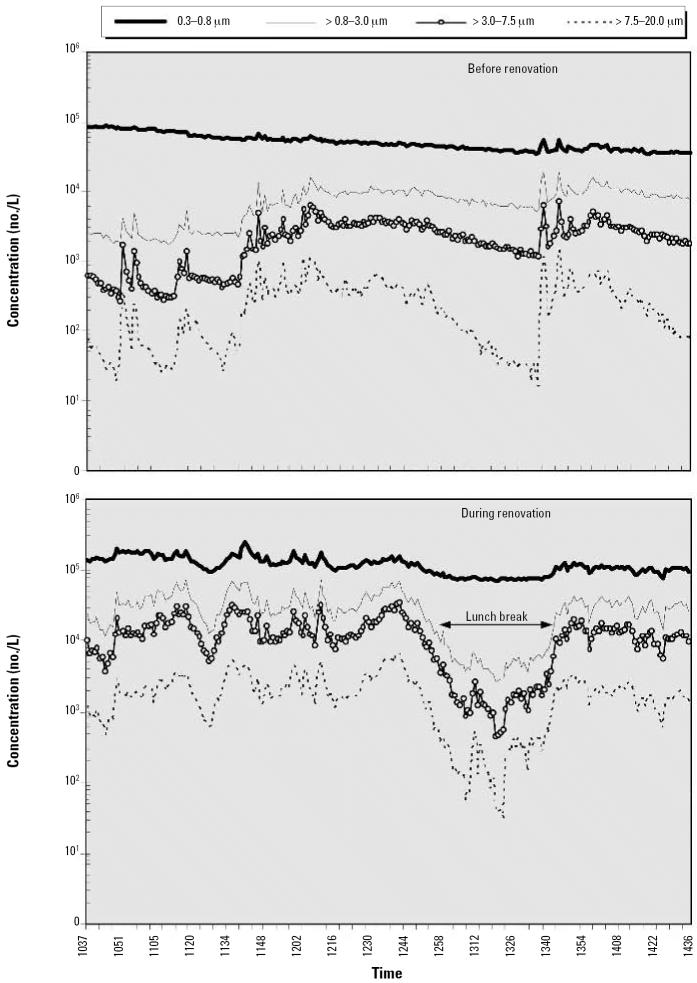
Size-selective particle concentrations before (*A*) and during (*B*) the renovation.

Figure 4 presents results of total particle counts, and Figure 5 shows size-selective particle concentrations. For the data analysis, we combined the 15 channels of the optical particle counter into four size ranges: 0.3–0.8, > 0.8–3.0, > 3.0–7.5, and > 7.5–20.0 μm. Results show that number concentrations of particles of all sizes increased during intervention, most clearly for particles > 0.8 μm. In contrast, during the lunch break, the concentration of particles > 0.8 μm decreased almost to the same level as measured before the intervention.

### Efficiency of respiratory protection

The WPF values for the two types of tested respirators are presented in Table 4. The average WPF against fungal spores for the elastomeric respirator was higher than that measured for the N-95 filtering facepiece respirator.

## Discussion

A critical finding of the present study is that in homes that held flood waters for several weeks, our flood cleanup techniques were associated with a reduction in mold and endotoxin levels. In all three homes, the interventions decreased mold levels and in some cases, the decreases were several orders of magnitude. In fact, the only measure that increased from pre-intervention to postintervention was endotoxin in house 2. This house had the most extensive flood damage, and during the intervention, endotoxin was higher than in the other houses. Because endotoxin is a cell wall component of gram-negative bacteria, we speculate that the bacteria were killed but that endotoxin remained in the settled dust after the intervention. Nonetheless, the levels of mold (as determined by three different methodologies) were drastically reduced after the intervention in house 2.

A strength of our study was the multi-pronged assessment of mold exposure. From our data, it is clear that the use of any one of the analytical methodologies (culture, microscopy, or PCR) would have shown that baseline levels of mold were high compared with agricultural, industrial, or other home environments. However, each analytical method gave different insight that we hope will inform future investigations in homes in New Orleans and in other environments. In contrast to culture methods to detect and enumerate fungi, spore microscopy and qPCR do not require viable fungal elements. In addition, spore microscopy and qPCR detect not only dead fungi but those that compete poorly on the various media used to grow fungi. The culture-based analyses (on both malt extract agar and DG18 agars) can underestimate the populations of some *Aspergillus* species by orders of magnitude when compared with qPCR (Meklin et al. 2004). In contrast, Basidiomycota spores (the common mushroom is in this fungal phylum) generally do not grow well on culture plates, and a probe was not available for PCR analysis; therefore, only spore microscopy enabled detection of this potentially allergenic group of molds (Horner et al. 1995; Lehrer et al. 1986). The low frequency of *Trichoderma* and *Alternaria* detection by PCR compared with the moderate to high frequency of recovery by direct microscopy and, to a lesser extent, culture likely reflects the airborne presence of taxa (e.g., *T. harzianum* and *Alternaria tenuissima*) for which PCR probes were not used or available. The spore microscopy also revealed that *Curvularia* was common in the houses. Although a PCR probe for *Curvularia* was not available for analysis of these samples, culture analysis should have been able to grow *Curvularia.* The low prevalence of culturable *Cladosporium* contrasts with the high prevalence, as determined by microscopy and PCR. *Cladosporium* is one of the most common fungal taxa recovered indoors and outdoors throughout the world (Chew et al. 2001, 2003; IOM 2004; Levetin 1995; Su et al. 2001). *Cladosporium* competes well with many of the other taxa that we recovered, so the reason for its decreased culturability in our samples remains elusive.

At baseline and particularly during intervention, we observed household levels of mold and endotoxin that equaled or surpassed those in wastewater treatment plants, cotton mills, and agricultural environments (Christiani et al. 1993; Lee J et al. 2006; Lee S-A et al. 2006; Spaan et al. 2006). Interestingly, the outdoor measurements were also higher than expected, ranging from 10 to 25 EU/m^3^. These exceed the average outdoor endotoxin measurements from 13 Southern California communities of 0.44 EU/m^3^ (Mueller-Anneling et al. 2004). In addition, the baseline levels of culturable mold in the three houses averaged 352,701 CFU/m^3^, which was much higher than the average (2,190 CFU/m^3^) or maximum level (48,760 CFU/m^3^) in homes sampled after the 1993 Mississippi River flood (Ross et al. 2000). The comparison with the Mississippi River flood study may somewhat overstate the disparity between the two floods because sampling in the earlier study occurred 1 year after the flood and only 40% of the residents in that study reported flood damage in their homes. Nonetheless, the levels of mold in the homes in the present study were extremely high. Given the level of visible mold observed when we first entered our demonstration homes, we were concerned about the level of respiratory protection required for entry and cleanup, and our measurements of both the mold levels and the respirator WPFs supported our concern.

By the time we planned the initial visit to house 3, we were able to characterize the particle sizes and conduct experiments to test the WPF of a disposable N-95 respirator and an elastomeric half-facepiece respirator. Our data indicate that counts of larger particles decreased drastically during the lunch break, taking almost 30 min to reach the lowest point, and reached counts of 50, 500, and 5,000/L of air (i.e., 50,000, 500,000, and 5,000,000 particles/m^3^) depending on the size range. These data, combined with the measurements of mold spores, culturable mold, and endotoxin, suggest that a substantial portion of the particle counts could be composed of fungal and bacterial material even during periods of inactivity.

The choice of respirator depends on the expected level of contamination. Currently, there are no threshold limit values for mold or endotoxin in the United States; however, a Dutch occupational health standard for endotoxin (50 EU/m^3^) existed for a brief time (Douwes et al. 2003). Also, excessive mold can be cited as a health concern by the Occupational Safety and Health Administration (OSHA) under their General Duty Clause (Occupational Safety and Health Act of 1970). Still, no defined level of mold is listed that warrants a specific level of respiratory protection. An assigned protection factor, which can be used in the initial selection of respirator type, gives the level of the respiratory protection that a properly functioning respirator or class of respirators would be expected to provide to properly fitted and trained users in the workplace (OSHA 2006). The assigned protection factor for both filtering facepiece and elastomeric half-facepiece respirators is 10 (e.g., 5,000,000 particles/m^3^ would be reduced to 500,000 particles/m^3^) (OSHA 2006). We found that, in the field, the WPF was lower for the N-95 respirator. In a previous study (Lee et al. 2005) in which the WPF against fungal spores was measured in agricultural environments, the geometric mean and geometric standard deviation of 21 WPF data points was 25 and 9.9, respectively. In addition, the WPF decreased with decreasing spore size. The values obtained from the N-95 respirator in house 3 were somewhat lower than those in agricultural environments. This could be due the composition of fungal load in the moldy building where small *Aspergillus/Penicillium* spores predominated. On the other hand, the WPF for the elastomeric respirator was clearly higher than the values obtained for the N-95 respirator in this study and in the previous agricultural study (Lee et al. 2005). Although the number of WPF data points in the present study is low, the results suggest that the elastomeric half-facepiece respirators offer at least 10 times the protection against fungal spores. Because the fungal spore concentrations were extremely high during the renovation, we question whether the protection offered by the N-95 filtering facepiece or the elastomeric respirators is sufficient. Furthermore, our WPF values are based on microscopic counting of intact spores. Recent studies have shown that exposure to fungi occurs also through sub-micrometer fungal fragments (Foto et al. 2005; Gorny et al. 2002; Green et al. 2005). These particles may penetrate at even higher rates as intact spores because the filter materials commonly used in N-95 respirators have maximum particle penetration of approximately 0.03–0.07 μm (Balazy et al. 2006).

The postintervention findings in house 1 highlight the critical importance of fully cleaning and drying a house. The upper walls and ceilings in the house were not vacuumed as part of the initial cleanup procedures. In addition, a small water leak saturated a portion of the concrete floor after work was completed. Because possessions remained inside, the house was closed and the humidity levels could have created a climate hospitable for further mold growth. The fact that culturable mold levels in this home were not substantially lower after intervention than before is likely related to these factors. After post-intervention sampling was completed, the water leak in house 1 was fixed and cleaning and mechanical drying was conducted.

House 1 also offers a cautionary note about the risks involved with leaving some of the original drywall in a home. Some flood cleanup guidance suggests that, in homes with minimal flooding, removing drywall on walls to 1.2 m (4 ft is the width of a standard sheet of drywall) instead of to the ceiling can save thousands of dollars in restoration costs (American Red Cross and Federal Emergency Management Agency 1992). However, many homes were submerged for weeks after Hurricane Katrina; although the water may have only wicked from the water line to the first 1.2 m, the homes were usually closed and the summer heat resulted in humidity levels similar to that of a terrarium. In our study, the house with the lowest water line (house 1) had visible mold growth in the wall cavities above 1.2 m after treatment. Although we cannot be certain that the growth would have occurred had cleaning and drying been adequate, the potential health risks of leaving the original drywall in the home must be taken into consideration.

The question of whether household bleach is an effective treatment mechanism was one of the most debated topics among the advisory group. Although it can have adverse environmental health effects, we used a dilute solution of bleach because of widespread concern of bacterial contamination and evidence that it could denature allergens (Chen and Eggleston 2001; Matsui et al. 2003) and possibly inactivate mycotoxins (Wilson et al. 2004). Bleach was selected primarily on the basis of federal guidance (American Red Cross and Federal Emergency Management Agency 1992; CDC 2005) and because it was widely accessible. In house 2, bleach was applied to the wooden building members, whereas it was not used in house 3. Because the postintervention findings for mold were similar in the two homes, we are encouraged that intensive dry cleaning followed by the application of borates appears to control mold growth. The use of dry cleaning without wet cleaning the wood had the added benefit of reducing the time of flood cleanup because the workers did not need to allow the wood to dry before applying borates. Research to examine alternatives to bleach is under way. If effective alternatives are identified, we would encourage their use to be incorporated into the federal emergency response protocols. Ideally the products should be accessible to consumers (both available and inexpensive) to enable their adoption.

There are several noteworthy limitations to the present study, including *a*) the small sample size; *b*) inconsistency of the number of samples collected; and *c*) possible lack of generalizability to other homes because of the home-selection process (Hung et al. 2005). The number of homes was necessarily small so that we could quickly try different types of cleanup procedures and assess their feasibility and efficacy. To conduct the interventions in a larger set of homes would have prevented expeditious reporting of findings. We had an inconsistent number of samples because of the lack of electricity; as of 22 July 2006, this was still a problem for much of New Orleans (Nossiter 2006), as was the lack of access to a fully functioning laboratory in New Orleans. We selected houses with a range of flood damage; however, the houses were typical New Orleans building structures, and the level of flooding was typical of many homes in the affected areas of New Orleans. The homes in Mississippi that were directly in the path of Hurricane Katrina sustained heavy wind damage, and we do not believe that our intervention results can be generalized to those homes. Nonetheless, our discussion of respiratory protection should be applicable to those homes with extensive mold growth.

The main goals for this pilot project were to synthesize, field test, and evaluate existing flood cleanup methods. For all houses, we removed ≥ 1.2 m of drywall, conducted HEPA vacuuming, used a borate salt solution to help prevent mold growth, and used bleach as a disinfectant. Using a variety of sampling and analytical methods, we observed airborne levels of mold and endotoxin, which often increased orders of magnitude during the intervention, and determined that workplace protection factors of some respirators can be suboptimal in such conditions. Although the generally accepted mold remediation protocols reduced bioaerosols in the demonstration houses, myriad issues including the qualifications of those performing the work (including homeowners), depth and duration of flooding, and the availability of electricity and supplies can affect the feasibility and ultimately the success of flood cleanup efforts. Our pilot project was not designed for determining whether the demonstration homes were safe for reoccupancy. Rather, we examined the extent to which homes that experienced significant and prolonged exposure to flood waters could be satisfactorily cleaned to enable reconstruction. Future research may include revisiting these homes after reconstruction to determine whether the low bioaerosol levels persisted or even continued to decline.

## Figures and Tables

**Table 1:** Baseline conditions and demonstration activities in houses.

	Baseline description	Removal of flood-damaged items	Cleaning	Drying	Biostatic agent used
House 1	Built in 1987 Two-story, slab on grade with attached apartment on the first floor Water line at 0.3 m above floor Roof had been patched with a temporary tarp before initiating flood cleanup Electricity operational	Owner moved some cleaned personal belongings (clothes, items in cardboard boxes) to the second floor of the home for storage Bottom 1.2 m of drywall was removed	Durable furnishings were wet-cleaned, wiped dry, and placed in storage inside the home Only concrete and vinyl floors and countertops were wet-cleaned Ceiling was not dry-cleaned	Owner left an upstairs ceiling fan on throughout the duration of the demonstration project, but windows were closed	Bora-Care (1:3 dilution)
House 2	Approximately 100 years old One-story raised home with plywood underlayment on 0.6-m piers Water line at 1.8 m above floor Electricity not operational	Furnishings and appliances were too damaged to salvage, so workers removed all furnishings for disposal Drywall was removed floor to ceiling	All surfaces were wet- and dry-cleaned	Windows left open for 2 weeks before applying a biostat treatment Windows left open after application of biostatic agent	Bora-Care (1:4 dilution) in front half of home Termite Prufe in back half of home
House 3	Built in 2004 One-story raised home with plywood underlayment on 0.9-m piers Water line at 0.6 m above floor Electricity not operational	Owners had removed all personal belongings, furniture, appliances, and lower kitchen cabinets prior to the demonstration Drywall was removed floor to ceiling	All surfaces were dry-cleaned Only toilets, sinks, bathtubs, and vinyl floors were wet-cleaned	Windows left open after application of biostatic agent	Termite Prufe

**Table 2:** Air sampling activities conducted for houses (indicated by X).

Activity	House 1	House 2	House 3
Indoor air (culturable fungi, endotoxin, PCR)			
Prework	X	X	X
During work	X	X	X
Postwork	X	X	X
Indoor air (fungal spores)			
Prework	X	X	X
During work	X	X	X
Postwork	X		
Outdoor air (culturable fungi, endotoxin, PCR)			
Prework			X
During work		X	
Postwork	X	X	X
Outdoor air (fungal spores)			
Prework	X	X	X
During work	X	X	X
Postwork	X		
Respirator-efficiency testing			X

**Table 3:** Baseline indoor concentrations of mold and endotoxin.

	Total culturable mold (CFU/m^3^)^a^	Total mold spore counts (spores/m^3^)	PCR results (spore equivalents/m^3^)	Endotoxin (EU/m^3^)
House 1	22,000–46,000	82,381	80,779	43
House 2	268,000–515,000	202,634	1,039,841	17
House 3	BR = 29,000–59,000	634,651	BR = 77,911	BR = 100
	LR/Kit = 332,000–342,000		LR/Kit = 178,067	LR/Kit = 139

Abbreviations: BR, bedroom; Kit, Kitchen; LR, living room.

a The average of samples grown on two different media and incubated at two different temperatures, resulting in four plates per sample collected.

**Table 4:** WPF against fungal spores with two types of respirators.

Respirator type	No. of WPFs	WPF GM (GSD)
N-95 filtering facepiece	2	5 (3.6)
Elastometric half-facepiece	4	40 (2.9)

Abbreviations: GM, geometric mean; GSD, geometric standard deviation. Each respirator type was tested in one subject.
